# Current Approaches, Typologies and Predictors of Deviant Work Behaviors: A Scoping Review of Reviews

**DOI:** 10.3389/fpsyg.2021.674066

**Published:** 2022-01-05

**Authors:** Salvatore Zappalà, Maha Yomn Sbaa, Elena V. Kamneva, Leonid A. Zhigun, Zhanna V. Korobanova, Anna A. Chub

**Affiliations:** ^1^Department of Psychology, University of Bologna, Bologna, Italy; ^2^Department of Psychology and Human Capital Development, Financial University under the Government of the Russian Federation, Moscow, Russia; ^3^Department of State and Municipal Administration, Russian Economic University named after G.V. Plekhanov, Moscow, Russia

**Keywords:** deviant workplace behaviors, counterproductive work behaviors (CWBs), work abuse, workplace deviance, abusive supervision, incivility at work, cyberloafing

## Abstract

This study provides a scoping review of the recent conceptual developments about the deviant work behavior and counterproductive work behavior constructs. It also examines the specific types of deviant work behavior that have been more consistently investigated in the last decade, and whether they cover the interpersonal or organizational type of deviant behavior. In addition, individual, group, and organizational predictors of deviant work behaviors are examined. A scoping review of reviews was conducted on Scopus and Web of Science databases and 54 studies published from 2010 to June 2021 were taken into account. Results show that more recent conceptualizations are based on well established models in the literature and consider the hierarchical structure of these two constructs. Recent reviews examine the relationships of deviant work behavior with job performance and ethical behavior constructs, the multilevel nature of deviant work behavior, and the consequences for the actor of the deviance. The specific types of deviant work behavior more frequently reviewed in the last decade are workplace abuse, incivility, ostracism, bullying and sexual harassment, and abusive and destructive leadership; this evidence suggests a much greater attention to interpersonal, rather than organizational, forms of deviant work behavior. Regarding antecedents, results show the continuing prevalence of personality factors antecedents. Limitations of the study and theoretical and practical implications for the field are also provided.

## Introduction

Treating colleagues rudely, assaulting or harassing subordinates, being absent or late at work, slowing down production, stealing the company’s money or materials are examples of workplace deviant behaviors. These and other types of deviant behaviors are commonly observed in workplaces and some studies estimated frequency and consequences of one type or another of deviant behavior. For instance, Porath (2016) reports that in the polls she conducted over 18 years, with thousands of workers worldwide, she observed that workers treated rudely by colleagues and/or customers rose from 55 percent in 2011 to 62 percent in 2016. A European survey observed that 11 percent of European employees are exposed to verbal abuse (Eurofound, 2016). Economic fraud perpetrated by employees cost companies about 6.3 United States dollar billion (Association of Certified Fraud Examiners [ACFE], 2016) or bullied employees are absent from work between 6 and 15 days a year (European Agency for Safety and Health at Work [EU-OSHA], 2010). Moreover, bullying costs British firms about 2 million pounds per year in addition to decreased employees’ creativity and organizational citizenship behaviors (Pate and Beaumont, 2010).

Despite evident differences in type, target and consequences, deviant behaviors share the breaking of some social, organizational, legal, or ethical norm. However, breaking organizational norms is not always harmful and employees’ behaviors may diverge from organizational norms for good reasons, such as, for instance, solving a client’s problem, implementing an innovative idea, or criticizing an incompetent superior to defend the interests of the organization (Narayanan and Murphy, 2017). Such positive deviant behaviors have been referred to as *constructive deviance* (Vadera et al., 2013) or *prosocial rule-breaking* (Morrison, 2006). However, although positive work deviance is gaining attention, it is not the focus of the current review and will not be considered in this study.

Negative behaviors dominate literature because of their variety and impact on both organizations and individuals. Such behaviors have been classified as *deviant work behaviors* (Robinson and Bennett, 1995), *counterproductive work behaviors* (Hollinger and Clark, 1983), but also *organizational misbehaviors* (Vardi and Wiener, 1996), *dysfunctional behaviors* (Griffin et al., 1998) or *workplace abuse* (Bowling et al., 2015). The first two terms, “Deviant Work Behaviors” (DWBs) and “Counterproductive Work Behaviors” (CWBs) are the ones that are used most frequently (Carpenter and Berry, 2017), interchangeably (Marcus et al., 2016) and in which “content overlap between measures of workplace deviance and CWB seems almost perfect” (Marcus et al., 2016, p. 205). Following this line of thought, we will use DWBs and CWBs interchangeably, although when citing authors, we used the term they utilized in their study.

Deviant work behaviors are defined as “voluntary behavior that violates significant organizational norms and in so doing threatens the wellbeing of an organization, its members, or both” (Robinson and Bennett, 1995, p. 556). Counterproductive work behaviors have, instead, been defined as “any intentional behavior on the part of an organization member viewed by the organization as contrary to its legitimate interests” (Sackett, 2002, p. 5). Both definitions cover a wide range of behaviors, such as theft, interpersonal violence, sabotage, being late, or wasting time at work, and many scholars have tried to capture what is common to such varied types of behaviors. Accordingly, overarching models have been developed to comprehend a broad set of workplace deviant behaviors. An example of this type of study is the typology of deviant behaviors developed by Robinson and Bennett (1995) or the structure of the counterproductive work behavior construct proposed by Sackett (2002). These studies examined multiple expressions of deviant work behaviors to figure out if they are “truly independent behaviors, with separate sets of antecedents” (Sackett, 2002, p. 6) or if, instead, they can be listed as components of a broader construct or, taking a step further, classified in a typology. For this reason, in this review we defined as “comprehensive studies” those studies that try to clarify, theoretically and/or methodologically, the broad construct of DWB, or CWB, or that examine the relationship between the broad DWB, or CWB, construct and the multiple forms of negative work behaviors subsumed under the broad constructs. Classifying different types of behaviors within the same category would give “parsimony and order to the diverse set of behaviors that comprise workplace deviance” (Robinson and Bennett, 1995, p. 557).

Another tradition of studies, however, is interested in just one single, specific, negative work behavior, such as theft (Hollinger and Clark, 1983), bullying (Einarsen, 2000), or, more recently, cyber deviance (Venkatraman et al., 2018). As a result, specific types of deviant behaviors, such as bullying (Einarsen, 2000), workplace incivility (Andersson and Pearson, 1999), or ostracism (Ferris et al., 2008), subsumed under the construct of DWB or CWB, have become an almost autonomous field of study because of the large knowledge they cumulated. We define studies focused on just one type of deviant work behavior, as “specific DWB” studies.

The aims of the present work are to review literature: (a) on the comprehensive constructs of DWB and CWB, and (b) on specific types of deviant work behaviors. To map recent literature in the field, we used a scoping review approach (Arksey and O’Malley, 2005; Peters et al., 2015), particularly a scoping review of reviews. Two main questions drove this study: (1) what are the more recent conceptualizations about the constructs of DWBs and CWBs? And (2) what are the specific types of negative behaviors that, within the DWB and CWB conceptualization, have been more extensively reviewed?

Clarifying these issues seems worthwhile for several reasons. From a theoretical point of view, it is important to ascertain if, building on previous comprehensive models [as that one developed by Robinson and Bennett (1995) or Sackett (2002)], more elaborated and extended typologies are available or if new, and different, conceptualizations have appeared. It is also important to examine if some specific type of deviant behavior has reached a level of maturity that is reflected in reviews that summarize the evidence. From a practical point of view, knowing which commonalities characterize DWBs provides a single perspective to cope with multiple harmful behaviors; alternatively, having a picture of the most developed and studied forms of deviant behavior should facilitate tailoring interventions that are better focused on specific deviant behaviors (Spector, 2006).

Considering that the domain of deviant work behavior comprehends a very large and heterogeneous literature (a recent review identified 91 terms addressing negative, deviant or counterproductive behaviors – Zhong and Robinson, 2021), we decided to conduct a review of reviews. In the following sections, we briefly describe the main models on the broad DWB and CWB constructs to highlight the debate about the various types of deviant behaviors and the dimensions, or categories, used to classify them. Then, we describe the scoping review methodology and report the main observed results.

### Conceptualizations and Dimensions of Deviant and Counterproductive Work Behaviors

Some scholars examined negative behaviors toward the organization, such as damaging machines or being late at work (Hollinger and Clark, 1983). Other scholars considered negative behaviors toward other persons, such as customers, colleagues, or subordinates. Accordingly, the distinction between Organizational deviant behavior and Interpersonal deviant behavior is well accepted and has received support in many factor analytic studies (Ones and Dilchert, 2013). In addition to these two targets, it has been suggested that a third target of negative destructive behavior can be the same employee, with self-destructive forms of deviance such as workplace drug or alcohol abuse (Martinko et al., 2002). However, although substance abuse is directed toward the self, it may impact both the organization, decreasing work performance, and other individuals, putting at risk colleagues and/or customers (Campbell and Wiernik, 2015).

According to Marradi (1990), using only one criterion (or *fundamentum divisionis*) to classify multiple objects originates a classification scheme; when several criteria are simultaneously used, then a typology is produced; and when several criteria are used in succession, across a series of classifications, then a taxonomy is produced [as when believer people can be distinguished in believers in divinities or spirits (animists) and the former in monotheistic or polytheistic, and so on]. In this regard, a well-known typology of deviant behaviors was developed by Robinson and Bennett (1995) using two dimensions. The two dimensions are (1) the target of the deviant behavior (the organization versus other persons) and (2) the consequences of the deviance (minor versus severe consequences). Crossing these two dimensions, four quadrants are identified: the first one, “production deviance,” identifies organizational deviant behaviors that have a minor impact, such as arriving late or leaving early from work, taking long coffee breaks, or intentionally working slowly. The second quadrant, defined as “property damage,” identifies organizational deviant behaviors with severe consequences, such as includes sabotaging, damaging equipment, or stealing materials from the company. The third quadrant, named “personal aggression,” identifies interpersonal deviant behaviors with severe consequences, such as harassing, abusing verbally or physically, or stealing from organizational members or customers. Finally, the fourth quadrant, named “political deviance,” concerns interpersonal deviant behaviors with minor consequences, such as making favoritism, gossiping about coworkers or supervisors, or hiding knowledge. To develop such typology, Robinson and Bennett (1995) used a multidimensional scaling approach, asking respondents to assess the similarity of 45 different deviant behaviors. Some years later, the two authors developed a self-report scale, the “workplace deviant scale,” to measure how often employees were involved in 7 interpersonal and 12 organizational deviant behaviors (Bennett and Robinson, 2000). This scale remains one of the most widely used instruments to measure workplace deviant behaviors (Ones and Dilchert, 2013).

A similar approach was followed by Gruys and Sackett (2003) who used factor analysis to summarize 66 different deviant behaviors in 11 types of counterproductive behaviors. A multidimensional scaling analysis found two dimensions again: interpersonal vs. organizational, and task related vs. task unrelated deviant behaviors. The 11 types of DWBs comprehend theft, destruction of property, misuse of information, misuse of time and resources, unsafe behaviors, poor attendance, poor quality of work, alcohol use, drug abuse, inappropriate verbal actions, and inappropriate physical actions.

Spector et al. (2006), building on different explanatory theories, proposed a five facets model of CWB: one facet, abuse, largely overlaps with the interpersonal deviance of Robinson and Bennett (1995) while the other four facets (production deviance, sabotage, theft, and withdrawal) concern organizational deviance. Subject matter experts were then asked to classify 45 CWBs in the proposed five facets. Hunt (1996) followed a different approach asking supervisors to assess how often subordinates engaged in deviant behaviors. Answers were grouped into five types of DWB: attendance, behavior unrelated or distracting from performance (e.g., unauthorized breaks or personal business during work time), unruliness, theft, and drug use. Finally, Sackett (2002) proposed a hierarchical model in which counterproductive behavior represents the top-level construct which includes middle level constructs [as, the four, five, or 11 types of deviant behaviors proposed, respectively, by Robinson and Bennett (1995); Hunt (1996), Gruys and Sackett (2003), and Spector et al. (2006)] which, in turn, include low-level constructs, which are the specific deviant behaviors, such as theft of cash, alter time card, misuse sick leave, arguing with customers, and so on.

The models just described used self-report questionnaires, containing brief statements of counterproductive acts that, once factor analyzed (or scaled), detected different types of behaviors (Marcus et al., 2016). Following Marradi (1990), such models range from the classification scheme of DWBs developed by Spector et al. (2006), to the typology which individuates four main types of DWBs on the basis of the two orthogonal dimensions, target and consequences of DWBs (Robinson and Bennett, 1995), to the three-level taxonomy by Sackett (2002). These models have the merit to summarize and map the multitude of deviant work behaviors in a shorter number of types of DWB. New perspectives considering DWB and CWB as umbrella terms are being introduced to better conceptualize the two constructs. For instance, Pearce and Giacalone (2003) presented a social perspective that considers the deviant behaviors implemented by teams and organizations.

In addition to these efforts to comprehend multiple types of DWB in a single framework, another tradition of studies focuses on just one type of DWB. Specific deviant work behaviors have been so extensively studied to become an almost autonomous field of study, such as bullying (Einarsen, 2000), workplace incivility (Andersson and Pearson, 1999) or ostracism (Ferris et al., 2008). However, technological and social changes occurring in workplaces lead to the appearance of new types of deviant behaviors. For instance, the wide adoption of the internet in workplaces made its use for non-work related activities quite common (Kim and Byrne, 2011). Initial companies’ efforts to block unauthorized use of the internet were nullified by the even wider availability of the internet on employees’ personal mobile phones (Jamaluddin et al., 2015; McBride et al., 2015). Even the increased use of remote work seems to result in new forms of deviant behaviors (Stich, 2020). Thus, although DWB is not a fresh knowledge, it is an evolving and growing construct that needs to be investigated with the passage of time and emerging global contextual factors (e.g., digitalization, globalization, and diverse workforce) that create new forms of deviant behaviors which may significantly affect the workplace (Baharom et al., 2017).

Well established is, instead, the distinction between Interpersonal vs. Organizational target of the deviant behavior. This distinction is present also in the CWB literature and is shortened in CWB Interpersonal (or CWB-I) and CWB Organizational (or CWB-O) (Ones and Dilchert, 2013). Examining classic studies, one might have the impression that, in previous studies, organizational deviance received more attention than interpersonal one. For instance, Bennett and Robinson (2000) questionnaire includes seven interpersonal and 12 organizational types of DWB; Spector et al. (2006) propose one interpersonal and four organizational types of deviant behavior; also the majority of behaviors mentioned by Hunt (1996) and Gruys and Sackett (2003) concern organizational deviant behaviors. However, considering the attention that in recent years was devoted to employees’ stress, wellbeing and organizational health (Cooper, 2011; Chen and Cooper, 2014) or decent work (Ferraro et al., 2015), one can wonder what is currently the balance between interpersonal and organizational deviant behaviors studies.

Predictors of DWBs are another interesting aspect in relation to studies that address DWB or CWB as broad, comprehensive, constructs, in comparison to studies that examine only a specific type of DWB. If the different types of deviant behavior share some commonalities, they should also share some predictor. However, this conclusion is questionable if, as observed in the meta-analysis by Berry et al. (2007), interpersonal and organizational deviant behaviors are highly correlated and have also differential relationships with personality factors and organizational citizenship behaviors. The issue is even more complex because literature reports a large variety of individual, group, and organizational level predictors of CWBs, such as, for instance, personality factors (Kolz, 1999), leadership and organizational culture (Goldman et al., 2006) or ethical climate (Appelbaum et al., 2005).

In short, some efforts have been made to classify the different types of deviant work behaviors resulting in comprehensive or overarching classifications and conceptualizations. At the same time, many studies conducted on specific types of deviant work behaviors (e.g., bullying) have accumulated so much knowledge to characterize them as almost an autonomous field of study and an autonomous phenomenon. Accordingly, the current paper aims to answer the following research questions: (1) have comprehensive models on deviant work behavior been proposed recently? Do they build and extend on previous models or present a new perspective compared to studies mentioned above? (2) which types of deviant behavior have accumulated enough knowledge to stand as an almost autonomous field of study with a growing body of reviewed literature? (3) are interpersonal and organizational deviant work behaviors equally represented in literature? (4) which are the main predictors of deviant work behavior at the individual, group, and organizational levels examined in the recent literature?

Given that DWB and CWB are very broad constructs that include many types of behaviors, and given that important reviews and meta-analyses have been conducted on the topic prior to 2010 (e.g., Sackett, 2002; Dalal, 2005; Berry et al., 2007; Hershcovis et al., 2007; Grant and Booth, 2009), to focus on more recent literature we decided to conduct a review of the reviews published on these two constructs between 2010 and 2021. In the following section, we describe the methodological criteria we used to conduct our scoping review.

## Methods

Grant and Booth (2009) acknowledged the great variety of available methods to summarize the evidence base and described the scoping review as one of 14 different types of literature review. Scoping reviews have a broad approach that aims “to map existing literature in a given field in terms of its nature, features, and volume… [They are] of particular use when a body of literature … exhibits a large, complex, or heterogeneous nature not amenable to a more precise systematic review” (Peters et al., 2015, p. 141). Such type of review addresses broad research questions (Arksey and O’Malley, 2005; Peters et al., 2015) and as our research questions are quite broad, we believe that a scoping review is particularly suited for our research questions.

Scoping reviews differ from systematic reviews especially for the aims (the latter aim to answer punctual questions often concerning effectiveness of interventions or treatments – Peters et al., 2015) while both methods share being systematic, transparent and replicable (Grant and Booth, 2009). Therefore, in our scoping review, we followed the guidelines for systematic reviews (PRISMA, Moher et al., 2009; Page et al., 2021). The search was conducted between April 2021 and July 2021 on Scopus and Web of Science Core Collection indexes (WoS). These two bibliographic databases are among the largest worldwide-used citation databases that analyze peer-reviewed literature (Zhu and Liu, 2020).

Considering the wide variety of constructs in literature related to DWB and CWB (Zhong and Robinson, 2021), our interest in comprehensive models, and the fact that, according to Sackett (2002), CWB and DWB are two top level constructs, we limited our search to reviews published after 2010 which included these two constructs, and their variations (e.g., deviance, deviant; behavior/s, behavior/s; work, workplace/s) in the title, abstract or keywords. This search was combined with the terms ‘review’ or ‘typol*’ in the title, abstract or keywords. The search strings are the following:

Scopus: [TITLE-ABS-KEY (devian* AND *work* AND behav*) OR TITLE-ABS-KEY (devian* AND workplace* AND behav*) OR TITLE-ABS-KEY (*counterproductive* AND *work* AND behavior*) OR TITLE-ABS-KEY (*counterproductive* AND workplace* AND behavior*)] AND TITLE-ABS-KEY (*review* OR typol*) AND PUBYEAR > *2009*;

Web of science: [(“devian* work behav*”) OR (devian* workplac* behav*”) OR (“counterproductive work behav*”) OR (“counterproductive workplac* behav*”)] AND (review OR typol*) Time span: 2010–2021 Indexes: SCI-EXPANDED, SSCI, A&HCI, ESCI.

**FIGURE 1:**
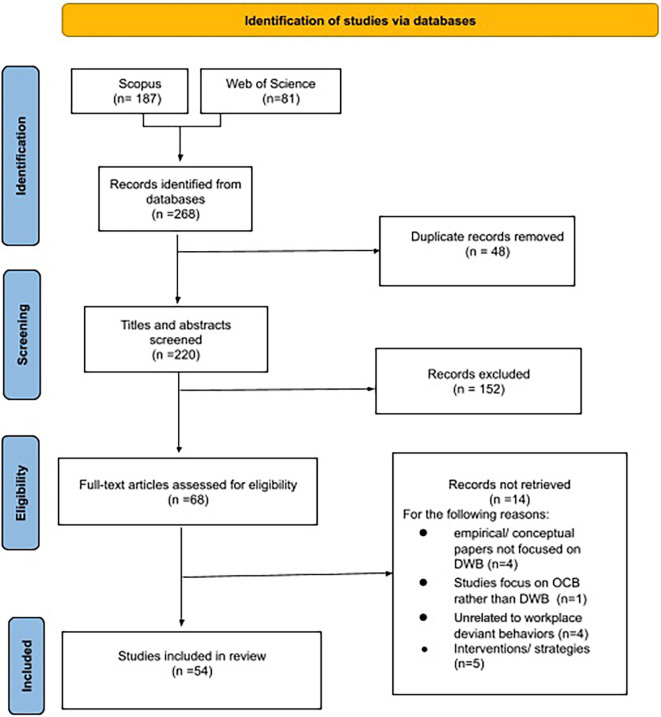
PRISMA flow diagram.

The articles that were screened had to fulfill the subsequent eligibility criteria: (a) published in peer-reviewed, scholarly journals; (b) literature reviews (systematic, non-systematic, or meta-analysis), thus single empirical studies were excluded; (c) written in English and not in other languages (as English language allowed all the authors to screen the studies and ensure more inter-rater reliability during the independent review and eligibility screening); (d) published between 2010 and 2021. Figure 1 reports the flow diagram of our search and elimination process and it shows that the preliminary search yielded 268 articles in total (187 records in Scopus and 81 records in WoS). After excluding duplicates (*n* = 48), 220 articles remained. To select primary studies for our scoping review, the first two authors independently screened title, type of study, keywords, and abstracts to examine whether the articles met our eligibility criteria. Most of the time, the two independent raters had high agreement on the eligibility of papers. In case of disagreement, a third author was asked to act as a third rater to check for the eligibility of the references based on the analysis of the full-texts and discussion among the three raters. Many records were excluded (*n* = 152) because they did not meet eligibility criteria (not in English language, empirical studies, book chapters or conference papers, validation of questionnaires, or topic unrelated to DWB because, for instance, pertaining to other disciplines, such as medical or clinical studies, law, or general psychology). After the screening, 68 articles remained. Then, the full text of these articles were thoroughly and independently examined by the first two authors. This resulted in the further exclusion of 14 articles because the full text clarified that, for instance, some articles were empirical or conceptual papers not focused on or unrelated to DWB, or concerned strategy to manage DWB. As a result, 54 articles were identified and included in this review. The following section describes the retrieved studies.

## Results and Findings

### Summary of the Results

Using the inclusion and exclusion criteria mentioned above, a total of 54 articles were selected for this review. Table 1 reports an overview of the retrieved studies. The first columns of the table provide preliminary information on author/s and publication year, type of study (systematic review, non-systematic review, and meta-analysis), and construct/s examined in each included review. The subsequent columns of the table are more analytic and serve to further classify the reviews based on our research questions. The column “scope of review” describes whether the review examined the broad or comprehensive constructs of DWB or CWB, or rather focused on a single type of DWB (e.g., bullying or ostracism). The next column further distinguishes the comprehensive reviews into (a) reviews that examined the broad construct of DWB in itself, for instance focusing on theoretical or psychometric aspects of the DWB construct (e.g., DWB measurement or level of analysis), and (b) reviews that examined the broad construct of DWB in relation to other variables or constructs, such as DWB and antisocial personality or DWB and abusive supervision. The former category of reviews is referred to as “Construct Conceptualization” (CC) while the latter one is referred to as “Interrelationship with other constructs” (IR). Finally, the remaining columns report whether the reviews examined interpersonal and/or organizational DWBs (“Type of DWBs”), and whether they considered individual, group or organizational antecedents of the DWB examined in that review (“Antecedents”), be it the broad DWB construct or a specific DWB (in other words, the antecedents of DWBs as a whole or, for instance, the antecedents of incivility).

**TABLE 1:** Overview of included studies.

Cd.	Author/s	Type of study Non-syst. rev. (N-SR), system. review (SR), meta-analysis (MA)	Construct/s	Scope of review Comprehensive (Com); Specific (Spec)	DWB construct conceptualization (CC) vs. DWB inter-relationsh. with other constructs (IR)	Type of DWB		Antecedents		
						**Interpersonal**	**Organizational**	**Individual-level**	**Group-level**	**Organizational-level**

1	Alias et al., 2013	N-SR	Deviant work behavior (predictors)	Comp.	IR			x	x	x
2	Appelbaum et al., 2012	N-SR	Workplace bullying (predictors and outcomes)	Spec		x		x	x	x
3	Bedi, 2021	MA	Ostracism (predictors and outcomes)	Spec		x		x	x	
4	Berry et al., 2012	MA	CWB measurement: self- vs. other report	Comp.	CC					
5	Bowling et al., 2015	N-SR	Workplace abuse (assessment, predictors, and outcomes)	Spec		x		x	x	x
6	Brooks, 2012	N-SR	Misbehavior (dimensions) and commitment in organizations	Comp.	CC	x	x			
7	Campbell and Wiernik, 2015	N-SR	Individual work performance and CWB as a dimension of it	Comp.	CC					
8	Carpenter et al., 2020	MA	Unit-level CWB (assessment, predictors, outcomes)	Comp.	CC	x	x		x	x
9	Cheang and Appelbaum, 2015	N-SR	Psychopath/antisocial personality disorder in leadership position	Comp.	IR			x		
10	Cortina et al., 2017	N-SR	Workplace incivility	Spec		x				
11	Cropanzano et al., 2017	N-SR	OCB and CWB according to social exchange theory	Comp.	CC	x	x			
12	DeSouza et al., 2017	N-SR	Ostracism targeted to LGBT employees	Spec		x				
13	Dhanani and LaPalme, 2019	N-SR	Vicarious workplace mistreatment	Spec		x		x		x
14	Faldetta, 2020	N-SR	Abusive supervision and negative reciprocity as antecedents to workplace deviant behaviors	Comp.	IR				x	
15	Farooq et al., 2021	N-SR	Types of bullying (vertical and horizontal)	Spec		x				
16	Ferris et al., 2017	N-SR	Ostracism and Incivility (comparison)	Spec		x				
17	Götz et al., 2019	SR	Deviance influenced by team and/or organizational norms	Comp.	CC				x	x
18	Grijalva and Newman, 2015	MA	Narcissism and CWB	Comp.	IR			x		
19	Hershcovis and Barling, 2010	MA	Effects of workplace aggression from different sources	Spec		x				
20	Howard et al., 2020	MA	Workplace ostracism (antecedents and outcomes)	Spec		x		x	x	x
21	Irum et al., 2020	N-SR	Workplace incivility and knowledge hiding	Spec		x		x	x	x
22	Jaikumar and Mendonca, 2017	N-SR	Effects of individual deviant behaviors on team members and team performance	Comp.	IR	x				
23	Kaushal et al., 2020	SR	Workplace ostracism	Spec		x				
						**Interpersonal**	**Organizational**	**Individual-level**	**Group-level**	**Organizational-level**
24	Klotz and Bolino, 2013	N-SR	OCB and CWB: a moral license perspective	Comp.	IR					
25	Koopmans et al., 2011	SR	Individual work performance and CWB as a dimension of it	Comp.	CC					
26	Krasikova et al., 2013	N-SR	Destructive leadership (antecedents and outcomes)	Spec		x	x	x	x	x
27	LeBreton et al., 2018	N-SR	CWB and dark triad personality	Comp.	IR			x		
28	Liao et al., 2021	MA	Four level un/favorable situational antecedents of CWB	Comp.	IR	x	x	x	x	x
29	Lugosi, 2019	SR	Deviant behaviors (antecedents and typologies)	Comp.	CC	x	x	x	x	x
30	Manning, 2020	N-SR	Organizational climate (compliance-based, integrity-based) and CWB	Comp.	CC	x	x			x
31	Marcus et al., 2016	MA	CWB (structure, dimensions, and models)	Comp.	CC					
32	Mercado et al., 2017	MA	Cyberloafing	Spec			x	x		x
33	Nair and Bhatnagar, 2011	N-SR	General model of antecedents and consequences of workplace constructive- destructive deviance	Comp.	IR	x	x	x	x	x
34	Narayanan and Murphy, 2017	SR	Workplace deviance (conceptual framework) and organizational climate	Comp.	IR	x	x			x
35	O’Boyle et al., 2011	N-SR	Multilevel models (organizational and group levels) of CWB	Comp.	CC			x	x	x
36	O’Boyle et al., 2012	MA	Dark triad personality and CWB	Comp.	IR			x	x	
37	Ong, 2012	N-SR	CWB as a dimension of job performance	Comp.	CC					
38	Phipps et al., 2015	N-SR	Big-five, impression management (IM) and CWB	Comp.	IR			x		
39	Piotrowski, 2018	SR	Dark triad, leadership and CWB	Comp.	IR			x		
40	Pletzer et al., 2020	MA	HEXACO personality and CWB	Comp.	IR			x		
41	Popovich and Warren, 2010	N-SR	Sexual harassment as a CWB	Spec		x				
42	Rakthin, 2015	N-SR	CWB and supervisor rating of employees performance	Comp.	CC					
43	Schilpzand et al., 2016	N-SR	Workplace incivility (experienced, witnessed and instigated)	Spec		x		x	x	x
44	Shockley et al., 2012	MA	State affect, discrete emotions, and job performance (CWB)	Comp.	IR			x		
45	Smith and Lilienfeld, 2013	N-SR	Psychopathy in the workplace and CWB	Comp.	IR	x		x		
						**Interpersonal**	**Organizational**	**Individual-level**	**Group-level**	**Organizational-level**
46	Spector, 2011	N-SR	Personality traits and CWB	Comp.	IR			x		
47	Stich, 2020	N-SR	Workplace stress, virtual office and CWB	Spec	IR		x			x
48	Sverke et al., 2019	MA	Job insecurity and performance (and CWB)	Comp.	IR					x
49	Tepper et al., 2017	N-SR	Abusive supervision	Spec			x		x	
50	Whelpley and McDaniel, 2016	SR	Self-esteem and CWB	Comp.	IR			x		
51	Wiernik and Ones, 2018	N-SR	(Un)ethical dimensions of CWB	Comp.	CC	x	x	x		
52	Wu and LeBreton, 2011	N-SR	Aberrant personality and CWB	Comp.	IR	x	x	x		
53	Zhang et al., 2019	MA	Abusive supervision as antecedent of CWB and OCB	Comp.	IR	x			x	
54	Zhong and Robinson, 2021	N-SR	Actor-centric consequences of deviant behaviors	Comp.	CC					

For the current scoping review we classified the retrieved reviews based on the content observed in each study. It is important to note that the reviews that were included in this study are very heterogeneous (i.e., meta-analysis, systematic reviews, and non-systematic reviews) and do not all belong to the same type (e.g., not all studies are meta-analyses). As mentioned above, they cover different topics (e.g., providing arguments and evidence about the multilevel nature of the DWB construct; meta-analyzing studies on the relationship between CWB and narcissism); consequently, the structure of the retrieved reviews and the type of information they provide are very different. In this sense, Table 1 and the present scoping review are different from a classical meta-analytic study which assesses the exact same type of information for each retrieved study (e.g., sample size, type of respondents, independent and dependent measures, or correlation). For this reason, some references reported in Table 1 are not assessed along all the criteria we established and present empty cells for the columns “Type of DWBs” and/or “Antecedents.” The reason is that those references did not contain that type of information. For instance, Koopmans et al. (2011) and Berry et al. (2012) examined the broad DWB construct but considered neither the interpersonal/organizational types nor the antecedents issue. Moreover, articles that mention DWB antecedents do not necessarily report on all the three levels outlined in the table. For instance, while Bowling et al. (2015) considered antecedents at the three levels, other scholars focused only on antecedents at one level (e.g., Cheang and Appelbaum, 2015) or two level (e.g., Carpenter et al., 2020).

Out of the 54 articles, there were 33 Non-Systematic Reviews (N-SR), 14 Meta-analyses (MA) and seven Systematic Reviews (SR). For what concerns publication year, 23 studies were published between 2010 and 2015, and 31 articles were published in the period between 2016 and July 2021. The comprehensive reviews, examining the broad construct of DWBs, were 36, while reviews on specific DWBs, such as bullying or cyberloafing, were 18. The comprehensive reviews were coded as Comp and the specific ones as Spec (see Table 1). The contents of comprehensive reviews were analyzed and distinguished in two groups as follows: 16 studies specifically concern the broad DWB construct (in Table 1, they are coded as CC), while 22 studies examined the interrelationships between the broad DWB construct and other variables, mainly as antecedents, but also as moderators or outcomes (in Table 1 coded as IR studies). Considering the specific type of DWBs, Table 1 shows that 16, out of 18, specific reviews investigated interpersonal DWBs (e.g., bullying, incivility, and ostracism) and only two reviews examined organizational DWBs (specifically, cyberloafing, or cyberdeviancy). When examining antecedents, 16 studies took into account DWB predictors at two or three levels (Individual, Group, and/or Organizational), while 18 reviews focused on just one level predictors of DWB: in this last case, 11 reviews described individual-level factors, three discussed group-level factors and four examined organizational-level factors.

**FIGURE 2:**
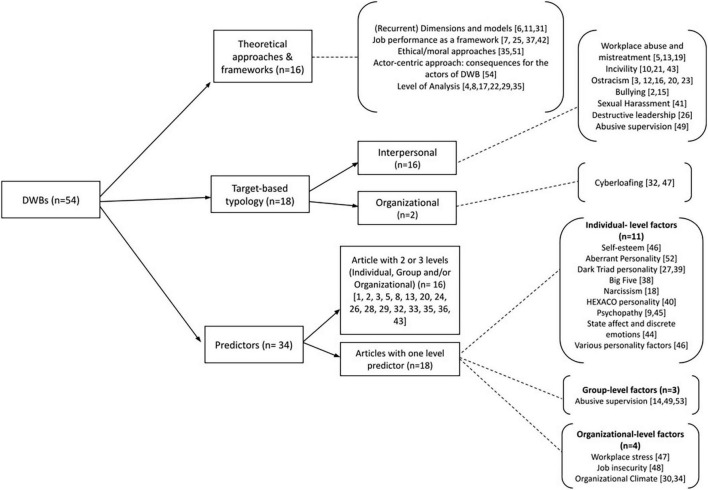
Retrieved articles clustered and mapped on the basis of topic. [*Note: Numbers represent article codes reported in* *Table 1*; *the three categories (Approaches, Typology, and Predictors) are not mutually exclusive; for this reason total is greater than 54*].

Figure 2 visualizes the studies included in our review and clusters them by the main topic. The three cluster of Figure 2 correspond to the three paragraphs of the Result section in which we answer our research question. In particular, Section “Comprehensive Approaches on Deviant and Counterproductive Work Behavior” answers to the question on conceptualizations on the broad DWB construct; Section “On Specific Forms and Types of Deviant and Counterproductive Work Behaviors” examines the specific types of CWB we retrieved distinguishing them in CWB-I and CWB-O; finally, Section “Predictors of Deviant and Counterproductive Work Behaviors” answers the question on the antecedents of DWB at individual, group and organizational levels.

### Comprehensive Approaches on Deviant and Counterproductive Work Behavior

#### (Recurring) Dimensions to Classify Deviant Work Behaviors

Two papers built on previous studies and models to discuss dimensions and internal structure of CWB (Brooks, 2012; Marcus et al., 2016), while another one (Cropanzano et al., 2017) used the social exchange theory to conceptualize CWB. Brooks (2012) proposed a four dimension model of CWB, with three dimensions belonging to previous classical models. The first dimension builds on Robinson and Bennett (1995) and is the person vs. organization target of CWB; the second dimension, following Gruys and Sackett (2003), concerns task relevance and distinguishes deviant behaviors related or unrelated to task activities (e.g., wasting working time that should be dedicated to the task vs. stealing or damaging company’s or colleague’s properties). The third dimension builds again on Robinson and Bennett and concerns the seriousness or harmfulness of the deviant acts; the fourth dimension, the quantity or amount of CWB, is necessary to quantify cases of individual CWB and does not refer to a specific CWB. Three out of the four proposed dimensions (except the quantity dimension) are then used to characterize different types of CWB, such as absenteeism, bullying, sexual harassment or theft.

Marcus et al. (2016) conducted a structural meta-analysis to examine the internal structure of the subdimensions, or types, of CWB proposed by three previous models (Robinson and Bennett, 1995; Gruys and Sackett, 2003; Spector et al., 2006). Different structural models were tested and results support a reflective structure of the CWB construct, which means that the various types of behaviors of the CWB construct (as, for instance, theft, or sabotage) are not independent but share a latent factor that underlies the various types of CWB. However, when more aspects of the different types of CWB (such as breath, content, modality or self-directed CWBs) are included in the model, then that underlying factor emerges less clearly. The review also concludes that Spector et al. (2006) model is not sufficient to cover the various types of CWBs, and that the interpersonal-organizational distinction should be enlarged to include self-directed CWB as a separate type of CWB. Self-directed CWB stands, in fact, as a different type of CWB to be distinguished, for instance, from safety violations because the latter can harm both the self and other individuals. Finally, the study distinguished between deviant behaviors that can affect only one target (e.g., only the organization, arriving late at work) and behaviors that can affect the organization and other individuals (such as stealing or damaging properties that belong to the company and/or to colleagues). The authors suggest that in this latter type of DWBs, antecedents, and interventions to fight the behavior toward one target (e.g., stealing from the company) are not the same and do not necessarily work with the behavior toward the other target (e.g., stealing from a colleague). This result contradicts the reflective nature, or the assumption of the general factor underlying the various DWBs; it seems also that the type of violation (e.g., stealing) is more important than variations in the target (the company or the colleagues).

Cropanzano et al. (2017) examined the deviant behavior construct within the frame of the social exchange between an actor (the organization, supervisor, or colleague) and a target (typically the employee). According to the theory, valence is an important criterion: the target typically will reciprocate the behavior or the hedonic value shown by the actor (e.g., justice is reciprocated with trust, or injustice is reciprocated with CWB). Cropanzano et al. (2017) add to the hedonic dimension the activity dimension: the actor can exhibit a positive vs. negative behavior but can also withhold a positive or negative behavior, suggesting that doing something negative is different from not doing something positive. The authors assume that employees will reciprocate both on the valence and the activity dimensions. Thus, an employee (or a target person) will show a high level of CWB in response to the actor’s active expression of undesirable, negative, behavior (e.g., high abuse); instead, the employee will reciprocate with low CWB the actor that withholds an undesirable, negative, behavior (e.g., low abuse). Other predictions are advanced for the other two conditions (active/desirable and inactive/desirable). Cropanzano et al. (2017) propose to consider situational constraints to explain why, despite the active undesirable behavior shown by supervisors or the organization, employees adopt an inactive (e.g., being late or wasting time) rather than an active (e.g., being aggressive or damaging machinery) CWB.

In summary, these three studies integrated the basic dimensions proposed by previous comprehensive models with dimensions to better map the multiple types of CWBs. They also suggest enlarging the dichotomy interpersonal-organizational by considering the specificity of self-directed CWBs and also taking into account the actor-target dynamic resulting from the combination of valence and activity dimensions.

#### Other Approaches to Deviant Work Behaviors: Job Performance, Ethical Behaviors, and Actor-Centric

Four papers examined CWB as a component of the higher-order job performance construct (Koopmans et al., 2011; Ong, 2012; Campbell and Wiernik, 2015; Rakthin, 2015). All of them report the proposal of Viswesvaran and Ones (2000) and Rotundo and Sackett (2002) that concluded that counterproductive work behavior (CWB) constitutes one of the three components of individual work performance, being task performance and Organizational Citizenship Behavior (OCB) the other two components. Ong (2012) reports that previous studies showed not only a negative relationship between CWB and OCB (employees that show deviant behaviors are less prone to help colleagues or suggest how to improve the job) but also a negative relationship between CWB and supervisors’ job performance ratings. Rakthin (2015) proposes that when CWB addresses the organization, and the supervisor is high in collectivism, employees’ job performance is even more unfavorably rated. Koopmans et al. (2011) observed that almost half of the generic work performance models, those that are valid for all types of work, include the comprehensive construct of CWB, with only few models considering severity or absenteeism/presenteeism dimensions of CWB. Koopmans et al. (2011) consider CWBs as a basic dimension of everyday individual work behavior. Also Campbell and Wiernik (2015), reviewing the major components of job performance, define CWBs on the basis of the interpersonal – organizational distinction. They add an “approach – avoidance” dimension, which distinguishes active actions against the organization/individuals (the approach type) and withholding, avoiding or staying away from the organization/individuals. It is evident here the similarity with the activity dimension proposed by Cropanzano et al. (2017). Finally, Ong (2012) considers task performance, OCB and CWB as components of the broad job performance construct, and describes CWB using dimensions (targets, consequences, and predictors) already established in literature.

Wiernik and Ones (2018) reviewed studies on the connection between CWB and (un)ethical behaviors. They argue that unethical behaviors concern violation of broader societal norms, while CWBs concern violation of organizational norms, and that unethical behaviors are related to actions mainly taken by managers, or other high-level staff, who abuse of their own power, while CWBs refer to actions mainly performed by low-level employees (such as absenteeism, theft or aggression). Despite such differences, Wiernik and Ones (2018) suggest that since unethical behaviors harm the organization’s legitimate goals, they should be considered as a component of the CWB domain. Wiernik and Ones (2018) refer, once more, to the interpersonal vs. organizational CWB distinction and compare different unethical and CWB typologies to support considering unethical behaviors as part of the broader CWB domain. The moral licensing theory is used by Klotz and Bolino (2013) to explain why some employees show both OCB and CWB. Their conceptual model predicts that employees with a high level of OCB, because they benefited the organization or colleagues, give themselves the permission to engage in a limited amount of immoral or deviant work behavior. When some activity, or task, they dislike appears, these employees will perform counterproductively without any fear of being considered bad persons because, in the past, they performed well.

Finally, although great attention has always been deserved to the victim of the deviant action, an interesting and recent perspective concerns the consequences that the deviant behavior has on the actor performing the action. Zhong and Robinson (2021) reviewed 110 articles that examined the consequences of DWB on an actor’s thoughts, feelings and subsequent behaviors. Five dominant theoretical perspectives are used by these authors to organize the actor-centric consequences of bad behaviors: affective, psychological needs, relational, psychological resources and cognitive dissonance perspectives. Zhong and Robinson (2021) conclude that the costs of negative actions seem to slightly prevail over the advantages they provide to the actor.

In conclusion, these studies show that the broad construct of CWB has complex interconnections which position it as an aspect of job performance and, at the same time, as an overarching construct subsuming unethical behaviors. The actor-centric perspective reviewed by Zhong and Robinson (2021) extends the conceptual domain of CWB.

#### Level of Assessment and Measurement of Deviant and Counterproductive Work Behaviors

Despite the significant progress, most research has tackled deviant work behaviors from an individual level of analysis, with limited focus on team and organizational levels (O’Boyle et al., 2011; Carpenter et al., 2020). O’Boyle et al. (2011) and Lugosi (2019) described multilevel models of CWB and underlined the influence that individual but also group and organizational factors have on the counterproductive behavior of individuals. The opposite approach was taken by Jaikumar and Mendonca (2017) that, in their integrative review, examined the effects that the negative behavior of a team member, such as withholding efforts, interpersonal deviance and negative affect, have on the other team members and on team performance.

Focusing on the deviant behavior of teams and organizations, Götz et al. (2019) argued that within organizations there are multiple reference groups which have different norms about what is an (in)appropriate behavior. Thus, breaking norms established at different levels requires antecedents at different levels. Reviewing 63 studies, Götz et al. (2019) highlighted external/societal antecedents (such as community violence) but also organizational (such as HR systems) and unit (such as team composition or team norms) antecedents of deviant behaviors perpetrated by units and within units.

Other, less usual, deviant actors are mentioned in the reviews proposed by Lugosi (2019) and Carpenter et al. (2020). Lugosi (2019) described the deviant behaviors carried out by organizations (such as the harsh treatment of employees or being silent about the risks of hazardous products), customers, and even suppliers. Instead, Carpenter et al. (2020) described deviant behaviors performed by teams, departments and organizations, and reviews unit-level antecedents. Adapting the definition of CWB proposed by Robinson and Bennett (1995), Carpenter et al. (2020) define CWB unit-level as “unintentionally and intentionally harmful behaviors perpetrated by the unit (e.g., team, department, and organization). These unit-level behaviors threaten the wellbeing of the unit, the organization, or both” (p. 4). Taking bullying as an example, the individual-level reflects the individual worker’s experience as perpetrator or target of bullying behavior; the unit-level captures the unit’s (e.g., team or department) collective experience with bullying which reflects how much bullying behaviors are displayed, accepted or discouraged within that unit. Social information processing is the theoretical framework used by Carpenter et al. (2020) to explain how the social environment influences unit-level DWB.

Two studies elaborated the measurement of CWB issue. Berry et al. (2012) noticed that multi-item measures have been developed to measure the broad construct of CWB, such as the Workplace Deviance Scale (Bennett and Robinson, 2000) and the Counterproductive Work Behavior Checklist (Fox et al., 2001). Such measures have been extensively used in self-report studies and also in other-report studies, in which supervisors and coworkers assess other employee’s CWB. Berry et al.’s (2012) meta-analysis, conducted on 40 studies, and 50 independent samples, showed that self- and other-reports of CWB are moderately correlated, and, with some exceptions, have similar correlations with common correlates. However, results showed that other-raters assessed target employees as less engaged in CWB than the self-raters reported being engaged in. These results suggest that CWB self-report seems to provide reliable results and that other-reports of CWB, difficult to be collected, add very little additional variance beyond that one explained by self-report. Unfortunately, individual CWB self-report has many threats to validity because of social desirability, lack of awareness of one’s negative actions, or fear of exposing or embarrassing oneself (O’Boyle et al., 2011). O’Boyle et al. (2011) proposed some methodological and statistical techniques to better measure CWB at the individual level and also measure group-level antecedents of CWB, such as group norms toward CWB or communication network and social relationships among team members.

In the following section, we report the contents of the retrieved reviews distinguishing the specific forms of interpersonal and organizational deviant behaviors on which they were focused.

### On Specific Forms and Types of Deviant and Counterproductive Work Behaviors

One of the most recurrent dimensions of DWB, proposed by Robinson and Bennett (1995), refers to the target: behaviors directed toward other people, and behaviors directed toward the organization. The reviews that were retrieved include workplace abuse, workplace incivility, ostracism, bullying, sexual harassment, abusive and destructive leadership, that we consider under the interpersonal CWB, and cyberloafing, considered under the organizational CWB.

#### Interpersonal Forms of Deviant Behavior

##### Workplace Abuse

Bowling et al. (2015) proposed workplace abuse as an “umbrella” concept, also used interchangeably with workplace mistreatment. They defined abuse as the physical and non-physical mistreatment, perpetrated by various persons in the workplace toward another person (Bowling et al., 2015; Dhanani and LaPalme, 2019). Workplace abuse subsumes many different types of interpersonal abuse in the workplace, such as incivility, bullying, interpersonal conflict, or ostracism. These behaviors include both physical and non-physical, active and passive mistreatment. What is also specific in this type of deviant behavior is that it can be carried out by different types of people in the workplace as, for instance the supervisor, coworkers, or subordinates of the victim and even customers. Additional information on abusive supervisors is reported in a later section.

Bowling et al. (2015) clarify that being an umbrella term does not imply that all the forms of abuse are interchangeable, but that those forms are conceptually and empirically distinct although functionally equivalent – because all of them aim to harm the victim – and for this reason, they are also positively related to one another. To test this idea, Bowling et al. (2015) sorted the 305 items of 15 different scales used to measure workplace abuse in 25 themes, such as yelling, doubting of competences, blaming, social isolation, supervisor abuse, invasion of privacy, property theft, threats of or actual physical aggression. The analysis of the 25 themes showed that, despite the variety, there is considerable overlap in the contents of the items, which suggests that although the scales measure different types of abuse, some themes are rather basic and are present across the different instruments.

Workplace abuse has a significant negative impact on the victims. For instance, it deteriorates the victim’s wellbeing and performance which, in turn, affects organizational functioning. However, Bowling et al. (2015) noticed that such relationships are complex: from one side, workplace abuse is a stressor and as such, it has a negative impact on victims’ performance. On the other side, abuse might promote an immediate increase in performance (but, probably, a decrease in the long term) when the victim improves performance because of the abuse from the supervisor. Finally, there might be a reversed causality. For instance, an extremely good or extremely bad performance might cause an abuse from other organizational members (Zapf, 1999). These conflicting possibilities might explain why the statistical negative relationship between abuse and job performance is not strong (Bowling et al., 2015).

In their systematic review, Hershcovis and Barling (2010) distinguished workplace aggression perpetrated by three different sources: supervisors, coworkers and outsiders. The authors argue that this distinction has major theoretical and practical implications because the magnitude of effects differs across sources. For instance, supervisor aggression may lead to employee job insecurity and lower levels of self-efficacy, while aggression stemming from outsiders (e.g., a patient against a nurse) is less likely to create job insecurity, but may lead to personal safety concerns. From a practical point of view, the organizational response to such threats should be tailored strategically. Results of their meta-analysis confirm that the aggression of the supervisor has the strongest effect on attitudinal and behavioral outcomes. In contrast, coworkers’ aggression had a stronger effect than outsiders on attitudinal and behavioral outcomes.

Although most workplace abuse and mistreatment studies have traditionally focused on the repercussions experienced by the direct victims, recent evidence suggests that workplace abuse can also affect “third parties” or bystanders who observe or become aware of others being abused (i.e., vicarious mistreatment). Dhanani and LaPalme (2019) highlight the processes through which vicarious mistreatment harms bystanders and the conditions under which this type of mistreatment has the strongest effects. Two main drivers generate this mistreatment: the organizational context, which allows negative behaviors and/or is characterized by high levels of stressors, and bystanders’ individual differences, with individuals more aware or sensitive to vicarious mistreatment. Dhanani and LaPalme (2019) also report that vicarious mistreatment affects job satisfaction and job performance of bystanders through emotional reactions.

More specific interpersonal CWBs are reviewed in the subsequent sections. Our results retrieved three reviews on workplace incivility, five reviews on ostracism, with one of them comparing ostracism and incivility (Ferris et al., 2017). Finally, two reviews on bullying and one review on sexual harassment complete the interpersonal section.

##### Workplace Incivility

A growing body of research investigates workplace incivility, defined as low-intensity deviant workplace behavior with an ambiguous intent to harm (Schilpzand et al., 2016). Being impolite, discourteous, or being personally attacked or humiliated in front of others are considered cases of workplace incivility. The majority of research on incivility tackles incivility instigated from coworkers but also supervisors and sometimes, even customers (Schilpzand et al., 2016; Cortina et al., 2017). Measurement of customers’ incivility is, however, infrequent. For instance, Workplace Incivility Scale (WIS; Cortina et al., 2001), the most used instrument to assess workplace incivility (Cortina et al., 2017), does not include customer-instigated incivility and it does not separate supervisor-initiated from coworker-initiated incivility.

Schilpzand et al. (2016) expanded the literature on workplace incivility distinguishing incivility with regard to the source (supervisor, coworker, or customer) and with regard to the type of incivility (experienced, witnessed, or instigated). Their review showed that most studies concerned experienced incivility (focused on outcomes of the uncivil conduct for targets), while the literature on instigated (focused on perpetrators’ characteristics as antecedents of perpetrators’ uncivil conduct) and witnessed (focused on negative outcomes for bystanders) incivility is substantially smaller than experienced incivility.

Repercussions of incivility mainly address victims’ work and non-work-related outcomes. This is clarified by Irum et al. (2020) that, in their review on workplace incivility, distinguish direct work-related outcomes (e.g., victims’ job performance) from indirect ones (e.g., job satisfaction, work withdrawal, and intention to quit). They also consider non-work-related outcomes which include stress, emotional exhaustion and work-life conflict.

Targeted employees’ responses to incivility have also been investigated. A common victim’s response, reported by Schilpzand et al. (2016), is self-blame. Victims are, in fact, more likely to hold themselves accountable for the mistreatment they receive; alternatively, they may implement some counterproductive response behavior. For instance, Irum et al. (2020) describe knowledge-hiding, a response recently investigated. The authors describe knowledge-hiding behavior as a reaction to incivility whereby knowledge is hidden by victims through playing dumb (i.e., pretending to not know what is asked), delaying the provision of information, providing incorrect information, or presenting rational explanations to justify not sharing the information. The rationale is that victims engage in knowledge-hiding, or more in general, on counterproductive behavior, to react to the uncivil actions perpetrated unto them. This may generate repercussions at various levels by decreasing individual task performance and also stalling organizational productivity at large.

##### Ostracism

While workplace incivility is considered a subtype of workplace mistreatment, workplace ostracism is considered a specific form of incivility (Howard et al., 2020) which includes behaviors such as being avoided at work, being shut out of conversations, or having one’s greetings going unanswered at work (Ferris et al., 2017). Ostracism has been defined from the victim’s perspective as well as the perpetrator’s perspective. Ferris et al. (2008) have defined the former as “the extent to which an individual perceives that he or she is ignored or excluded by others” (p. 1348). The latter has been defined by Robinson et al. (2013) as “when an individual or group omits to take actions that engage another organizational member when it is socially appropriate to do so” (p. 206).

Ferris et al. (2017) framed ostracism as a form of workplace incivility because it fits the criteria associated with incivility previously outlined: ambiguity (e.g., if an individual does not return someone greetings at work, it is not clear if he/she is ostracizing or simply failed to hear the other person) and low intensity (e.g., abstaining from conversing with someone). However, the authors suggest that while ostracism represents the non-interactive component of incivility (where perpetrator and victim of ostracism do not interact, neither positively nor negatively), incivility represents the interactive component (perpetrator and target of incivility interact, usually in a negative manner). Therefore, scholars (Ferris et al., 2017; Bedi, 2021) argue that the lack of interaction is the main way to distinguish ostracism from other forms of workplace mistreatment such as incivility, bullying or any other form of workplace abuse. Kaushal et al. (2020) further illustrated specific behaviors that reflect ostracism, such as linguistic ostracism (when a conversation is conducted using a language that others around cannot understand), social rejections (when a person tries to build a relationship or an alliance with someone else that instead refuses such connection and avoids any social contact), or organizational shunning (when a person that once was part of a group is systematically excluded by rites or rituals that underline organizational membership).

Although ostracism may seem harmless, ostracized employees are strongly affected. Ostracism is a threat to the sense of belonging (Howard et al., 2020). As social beings have a fundamental and innate need for belonging, individuals tend to recognize any small hint of ostracism, which explains the strong psychological impact that ostracism has on individual wellbeing (Howard et al., 2020). Studies clearly confirm that victims of ostracism are caught in endless rumination associated with anxiety, anger, or sleep interference (Howard et al., 2020; Bedi, 2021). This can be even more true for stigmatized groups (i.e., minorities). In their review, DeSouza et al. (2017) state that many studies show LGBT employees reporting feeling ostracized in their workplaces. These manifestations are either due to coworkers who exhibit sexual prejudice or to the systematic heterosexism in their organizations. The authors further illustrate that if policies or norms utilize heterosexist language for same-sex partners (e.g., referring to a heterosexual employee’s romantic partner as “spouse” but referring to an LGBT employee’s partner as a “friend” or “roommate”), it may cause further withdrawal of LGBT employees.

Other negative effects on wellbeing were also reported in the meta-analysis by Bedi (2021) who showed that ostracized employees tend to suffer from reduced self-esteem and job satisfaction, and increased turnover intentions, workplace deviance, and emotional exhaustion.

##### Bullying

Our results retrieved two short reviews on bullying. Appelbaum et al. (2012) used the bottom right quadrant of Robinson and Bennett’s (1995) classification (where interpersonal and severe behaviors occur) to frame bullying. Bullying lies within the spectrum of interpersonal deviant workplace behavior that ranges from inappropriateness to homicide. Einarsen (1999) defined workplace bullying as “the repeated actions and practices that are directed to one or more workers, which are unwanted by the victim, … but clearly cause humiliation, offense, and distress, and that may interfere with job performance and/or cause an unpleasant working environment” (p. 17). Typical manifestations of perpetrated bullying are verbal abuse, physical or non-verbal threats, intimidating or humiliating, work sabotage that interferes with the work performance of the victim, exploitation of a physical, social or psychological vulnerability of the victim, or some combination of these types of behaviors.

In their review, Farooq et al. (2021) distinguished between vertical (i.e., supervisor-to-subordinate) and horizontal (i.e., colleague-to-colleague) bullying. While vertical bullying takes the form of abusive behaviors directed at the subordinate (e.g., over-burdening the subordinate with work), horizontal bullying includes peer-to-peer forms, like not cooperating with a new employee or harassing colleagues.

Bullying affects both targeted employees and organizations as a whole (Appelbaum et al., 2012). For instance, bullying is directly correlated with low job satisfaction, high employee turnover, increased absenteeism and decreased organizational commitment. From an organizational standpoint, bullying can also harm a firm’s performance by shattering the job-related abilities of victims (e.g., motivation, task learning, and team interdependence). The authors argue that workplace bullying is mostly determined by the work environment and Farooq et al. (2021) report that there are three main ethical qualities that could control bullying in the workplace: humaneness, respect, and decency. Taken together, establishing an ethical climate at work seems to be an effective method to limit workplace bullying (Appelbaum et al., 2012; Farooq et al., 2021).

##### Sexual Harassment

Our review retrieved one study that explicitly presents sexual harassment (SH) as a counterproductive work behavior (Popovich and Warren, 2010). In fact, SH meets the criteria presented in most definitions and theories concerning CWB: inappropriate verbal or physical actions, such as making unwanted sexual advances toward a coworker, subordinate or customer. Following Robinson and Bennett (1995) typology, Popovich and Warren (2010) framed SH as a form of personal aggression. However, Popovich and Warren (2010) report that SH overlap with other types of CWBs as, for instance, workplace aggression, emotional abuse, bullying and even revenge. Moreover, SH, as other interpersonal CWBs, shares the characteristics of the subjective perception of the target and the ambiguity of intent which makes it more difficult to diagnose and assess it.

Interestingly, Popovich and Warren (2010) presented SH as a function of power and extended it by describing the bases of power across individual, organizational, and societal levels. At the individual level, the abuse of power is when a male supervisor makes sexual advances to a female subordinate, indicating that her ability to keep her job depends on her compliance with his demands. At the group or organizational level, this reflects the creation of an environment in which female subordinates feel obliged to accept sexual jokes and other comments by those who have formal power over them. It is noteworthy to mention that SH incidents may also involve social agents from outside the organization, representing a societal level of influence.

##### Destructive Leadership

Our literature search provided one study that focuses on cases of interpersonal deviant behaviors where the perpetrator holds a supervisor or leadership position. Krasikova et al. (2013) define Destructive Leadership (DL) as “volitional behavior by a leader that can harm or intends to harm a leader’s organization and/or followers by (a) encouraging followers to pursue goals that contravene the legitimate interests of the organization and/or (b) employing a leadership style that involves the use of harmful methods of influence with followers, regardless of justifications for such behavior” (p. 1310). Despite this definition that outlines DL as a form of CWB, the authors finely distinguish between DL and CWB. The following example illustrates their distinction: a manager stealing from the organization is not a destructive leader, rather s/he is performing a deviant behavior; instead, a manager directing his or her followers to steal is an example of a destructive leader. Therefore, DL involves harmful actions performed by leaders in the process of leading followers toward certain goals, whereas CWB involves harmful actions that do not involve leading others. Krasikova et al. (2013) argue that DL process arises when the leader chooses to pursue a goal that can harm the wellbeing of the organization, like seeking personal wealth at the expense of organization’s earnings; however, it also arises when the leader chooses to pursue a goal, organizationally sanctioned or not, in a way that can harm the wellbeing of the followers (for instance, bullying them). In this latter case, DL can be interpreted as interpersonal CWB targeted toward employees.

##### Abusive Supervision

Abusive supervision is a form of workplace incivility instigated by supervisors toward subordinates, which includes uncontrolled outbursts, inappropriate blaming, or public ridicule (Tepper et al., 2017). In their review, Tepper et al. (2017) report that between 2011 and 2015, more than 150 papers were published investigating the relationships between abusive supervision and its presumed antecedents and consequences. Hence, several terms have been used in the literature to express abusive supervision, such as supervisor aggression or petty tyranny. However, abusive supervision, a concept introduced by Tepper (2000), seems to be the most frequently used. Research shows that workplace abuse coming from supervisors seems to have a greater impact than when it comes from other coworkers (Tepper et al., 2017). However, perceptions of abusive supervisor behavior are more likely to create inter-subordinate disagreement, which reveals the subjective perceptions and experiences of the targeted subordinates.

In their review of literature, Tepper et al. (2017) conclude that although exposure to abusive supervision is rare and seem to involve a limited part of the workforce (about 10% of it), it is nonetheless associated with several dysfunctional outcomes, such as employee withdrawal, turnover, deteriorated employee wellbeing and also higher levels of subordinates’ CWBs. Evidence suggests that abusive supervision, as coworker incivility, generates subordinate CWB through a decrease of affective commitment, interactional justice, and perceived organizational support, and an increase of emotional exhaustion.

#### Organizational Forms of Deviant Behavior

Although abusive or destructive leadership could also be considered as a way of expressing organizational deviance, we found only two papers (Mercado et al., 2017; Stich, 2020) that expressly examined some forms of deviant behaviors targeting the organization and facilitated by information technology. The great diffusion of information technology is making it easier for employees to use organizational and personal devices to connect and navigate the internet, using social media, sending personal emails, or playing and gambling online. All such behaviors go under the term of cyberloafing, and many managers are worried about employees’ waste of time, decrease of productivity and unsafe behaviors.

The meta-analysis conducted by Mercado et al. (2017) on 54 independent samples showed that, despite expectations and against stereotypes, age, job tenure and organizational level seem unrelated to cyberloafing. Some broad personality variables, such as emotional stability, conscientiousness, and agreeableness, exhibited modest negative relationships with cyberloafing, whereas self-control and self-efficacy demonstrated a strong and noteworthy negative relationship with cyberloafing. Interestingly, although cyberloafing was strongly and positively related to overall CWB, and in particular to time theft, its relationship with job performance was negligible. Mercado et al. (2017) report that some scholars consider cyberloafing as a form of CWB, while other scholars suggest that it may be considered more positively as an opportunity to decrease the stress experienced during work time or to compensate the time dedicated to work during off-hours time. Similarly, Stich (2020) reports that some scholars consider cyberloafing a coping response to workplace stress experienced by employees during telework. In addition, Stich (2020) suggests that virtual work offers the possibility to show two types of deviant behavior: using the internet for non-work-related activities, such as navigating the internet for personal information or online gaming, and more severe forms of deviant behaviors such as stealing data, hacking internal or external infrastructures, or colleagues’ and supervisors’ accounts.

### Predictors of Deviant and Counterproductive Work Behavior

Most of the theoretical writings on why people engage in CWB have adopted a personological point of view, focusing on individual differences (Wu and LeBreton, 2011) and personality (O’Boyle et al., 2011; Carpenter et al., 2020) and neglecting organizational factors, such as organizational climate or leadership (O’Boyle et al., 2011). This trend appears to have remained stable over the past 10 years. Relying on the traditional tripartite classification of individual, team, and organizational factors affecting individual work performance, our results reveal that ten studies described antecedents of deviant work behaviors located at the three levels of individual, interpersonal/group, and organizational level and six studies described antecedents of CWB at two levels (e.g., individual and group antecedents). There are 18 studies focused on just one level antecedent: ten studies concern individual-level antecedents, three studies only group-level, and four studies only organizational-level antecedents of CWB (see Table 2).

**TABLE 2:** Antecedents of workplace deviant behaviors, distinguished by level of antecedents and type of deviant work behaviors.

Antecedents	Workplace deviance (CWB in general) (Nair and Bhatnagar, 2011; O’Boyle et al., 2011, 2012; Alias et al., 2013; LeBreton et al., 2018; Lugosi, 2019; Carpenter et al., 2020; Liao et al., 2021)	Workplace abuse (Bowling et al., 2015; Dhanani and LaPalme, 2019)	Incivility (Schilpzand et al., 2016; Irum et al., 2020)	Destructive leadership (Krasikova et al., 2013)	Ostracism (Howard et al., 2020; Bedi, 2021)	Bullying (Appelbaum et al., 2012)	Cyberloafing (Mercado et al., 2017)
Individual-level	Age (−), Education (−), Gender [Male (+)]; Type of contract, seniority (−) Negative affectivity (+) Agreeableness (−); Conscientiousness (−); Emotional intelligence/stability (−); Anger (+); Idealism (−); Dark triad (narcissism, Machiavellianism, and psychopathy) (+); Job satisfaction (−); Org. commitment (−)	*Perpetrators’* personality [agreeableness (−), neuroticism (+), conscientiousness (−)]; Victims’ demographic characteristics [group membership (+), mistreated in family (+), agreeableness (-), neuroticism (+), negative affectivity (+), impulsivity (+)];	Conscientiousness (−); Negative affectivity (+); Dominating conflict management style (+); Target’s social (age, race, obesity) and personality (disagreeable, neurotic) characteristics (+)	Leader’s biased information processing (+); Tendency to emphasize self-interest (+); Machiavellianism, narcissism, and psychopathy (+); Impaired self-regulation (+); Negative trait affectivity and paranoid tendencies (+); Leader’s perceptions of goal blockage (+)	Negative affectivity (+); Positive affectivity (−); Big Five: conscientiousness (−), agreeableness (−), extraversion (−), proactivity (−), neuroticism (+). Political skills (−). Social skills (−). Need to belong (−); Future orientation (−)	Psychopathic traits of leaders (+)	Level of education (+). Big Five trait personality: agreeableness (+), conscientiousness (+), and emotional stability (−). Self-control (−) and self-efficacy (+). Perceptions of inequity (+); Perceived benefits of cyberloafing (+).
Group-level	Abusive leadership/supervision (+); Ethical leadership (−), Transformational leadership (−); Quality of supervision (−); Team level attitudes (−); Team level justice perceptions (−); Team level negative affectivity (+); Pressures to conformity (+); Team dissimilarity (+); Team cohesion (−); Coworker aggression and conflict (+)	Social discrimination toward members (+)	Passive leadership (+); Ethical and charismatic leadership (−); CWB of the target (+)	Incompetent, unmotivated, or intentionally uncooperative followers (+)	Abusive supervision (+). LMX (−). Perceived social support (−).		
Organizational/work level	Ethical climate (−); Organizational justice (−); Perceived organ. support (−); Organizational constraint (e.g., restrictive rules, lack of resources) (+); Trust in management (−); Breaching psychological contract (+); Work stress (+); Strategic HR manag. (−); Badly designed tasks/processes (+); Overly prescriptive/absent rules (+); Misleading/non-existing guidelines on acceptable behaviors (+); Job/role ambiguity (+); Surveillance/control (+)	Climate tolerating mistreatment (+); work stress (+); Role ambiguity (+); Role conflict (+); Organizat. constraints (+); Job autonomy (−)	Power and status dynamics (+); Work stress (+) Psychol; contract breach (+); Norms for civility (−); Role ambiguity (+) Role conflict (+) Distributive injustice (+)	Scarcity of resources for leaders’ goal achievement (+); Acceptance of harm-doing (+); Acceptance of destructive leadership as effective way to achieve goals (+)	Workplace incivility (+)	Work stress (+). Time pressure (+). Work uncertainty (+).	Hours worked per week (+). Job autonomy (−). Boredom (+). Employee engagement (−). Organizational justice perceptions (−). Technology access (+) (proportion of time spent on the internet).

*Note: Table includes antecedents reported in reviews citing at least two level predictors (i.e., individual, group, and/or organizational levels). The sign (+ ⁣/⁣−) expresses the direction of correlation between the antecedent and DWB.*

Table 2 lists the antecedents reviewed in studies that took into account antecedents at two or three levels. The second column of Table 2 lists predictors mentioned in studies that considered the broad construct of deviant or counterproductive work behavior, while the other columns consider predictors of specific deviant behaviors. Most common individual level antecedents include socio-demographic characteristics as well as personality traits of perpetrators and victims of deviant behaviors (e.g., O’Boyle et al., 2012; Bowling et al., 2015; Schilpzand et al., 2016; Lugosi, 2019; Howard et al., 2020). The most common group antecedents of CWB include negative leadership styles (such as abusive or passive), that perpetrate, approve or ignore deviant behaviors, and team norms and processes that create a social context in which deviant behavior is considered as an acceptable one (e.g., O’Boyle et al., 2011; Krasikova et al., 2013; Carpenter et al., 2020; Bedi, 2021). Finally, organizational level antecedents are, among others, workplace stress, job insecurity, role ambiguity, perception of organizational injustice, ethical climate and organizational procedures that do neither challenge nor sanction inappropriate behaviors (e.g., Appelbaum et al., 2012; Alias et al., 2013; Lugosi, 2019; Irum et al., 2020). In the following sections, we describe the antecedents considered in those reviews that examined only one level antecedents.

#### Individual-Level Antecedents

Most of the retrieved reviews that addressed exclusively individual antecedents were focused on personality factors, in particular Big Five and the Dark Triad model, thus confirming the personological perspective on CWB (O’Boyle et al., 2011; Carpenter et al., 2020).

Phipps et al. (2015) reviewed the literature on Big Five, OCB, and CWB and report that conscientiousness is the personality dimension with the most significant negative correlations with CWB. This result was observed across studies conducted in different countries, across sectors and professions. Pletzer et al.’s (2020) meta-analysis took into account 29 studies that used HEXACO, a six personality dimension model, and found the same result. In addition, they also found that narrow facets or subcomponents of the broad dimension (e.g., conscientiousness is composed by four facets: diligence, organization, perfectionism, and prudence) have higher criterion validity for deviant behavior than the broad dimensions. The authors concluded that it should be possible to predict deviant behavior using the narrow facets because they are more parsimonious description of personality.

Following a similar approach, Wu and LeBreton (2011) reviewed literature on the three traits, narcissism, Machiavellianism, and psychopathy, known as the dark triad or the aberrant personality. The authors concluded that the dark triad can be a fruitful approach to understand the dispositional basis of CWB. One year later, O’Boyle et al. (2012) published a meta-analysis on 245 independent samples observing that increases in CWB were related to increases in two components of the dark triad, narcissism and Machiavellianism, with narcissism being the stronger predictor of CWB. In addition, such positive associations were moderated/weakened by two variables: the role position of leadership and the in-group collectivistic culture. In the first case, CWB is decreased because leaders have to conduct the team and, thus, have to mitigate their narcissism and Machiavellianism. In the second case, collectivist teams are less likely to tolerate the manipulative and disrespectful actions of individuals high in dark triad components.

The positive relationship between narcissism and CWB was tested by Grijalva and Newman (2015) on a larger sample of studies. These authors confirmed that narcissism remains the largest predictor of CWB even after controlling for the Big Five personality traits. In addition, Grijalva and Newman (2015) conducted an international study that confirmed O’Boyle et al. (2011) conclusion that the in-group collectivistic culture moderates the narcissism-CWB relationship. The literature review by LeBreton et al. (2018) suggest that two mediators (perceptions of organizational politics and perceived accountability) and four potential moderators (political skill, organizational transparency, organizational policies, and organizational culture/climate) affect the relationship between Dark Triad traits and CWBs.

The effects of another component of the dark triad, psychopathy, were examined in the reviews by Smith and Lilienfeld (2013); Cheang and Appelbaum (2015), and Piotrowski (2018). In particular, Smith and Lilienfeld (2013) checked the results of a previous study in which the presence of a psychopathic leader was associated with a higher frequency of CWB and aggressive behaviors. Their meta-analysis showed that leaders’ psychopathy was negatively associated with job performance and positively with CWB. However, both effect sizes were so small in magnitude (respectively −0.07 and 0.07) that Smith and Lilienfeld (2013) concluded that such association should be tested with more rigorous studies. Cheang and Appelbaum (2015) conclude that two characteristics of psychopathy (lack of empathy and high level of manipulation skills) contribute to aversive managerial actions (such as partiality or breaking of promises) that may generate employees’ deviant behaviors. Even the 22 studies examined by Piotrowski (2018) suggest that the relationship between dark triad personality and dysfunctional leadership behaviors toward subordinates contribute to employee’s expression of abuse and incivility.

Two studies examined the relationship between specific personality dimensions and CWB. In particular, Spector (2011) proposed that some personality variables, such as narcissism, negative affectivity and angry personality, influence the cognitive representation and the emotional reactions to environmental events that lead to CWB. Other personality variables, i.e., locus of control and self-control, instead, inhibit the CWB. A meta-analysis, conducted on 21 samples, found a correlation between self-esteem and CWB of −0.26 (Whelpley and McDaniel, 2016). Another meta-analysis, on 114 independent samples from 98 studies, observed that individuals with a disposition to react with positive emotions have higher scores on task performance and OCB and lower scores to CWB, while the opposite is observed for individuals with relatively stable negative emotions (Shockley et al., 2012).

#### Group-Level Antecedents

The three studies that describe group-level antecedents of DWBs concern abusive supervision. Evidence suggests that abusive supervision decreases subordinates’ affective commitment, interactional justice, and perceived organizational support, as well as increases emotional exhaustion that, in turn, contributes to subordinates DWB (Tepper et al., 2017). Even the review by Faldetta (2020) shows that abusive supervision causes employees’ feelings of injustice, which motivate them to seek revenge performing some type of deviant behavior. Negative reciprocity theory, which states that individuals return negative treatment for negative treatment, is used to explain that process (Faldetta, 2020).

In a structural equation model meta-analysis, conducted on 427 primary studies, Zhang et al. (2019) observed that both work stress and perception of organizational justice mediate the relationship between abusive supervision and CWB, and that work stress explains a greater proportion of this effect in comparison to organizational justice.

#### Organization-Level Antecedents

Multiple organization-level antecedents of CWB have been investigated. Alias et al. (2013) reviewed studies that confirm that when ethical climate, organizational justice, and perceived organizational support are insufficient or inadequate, CWB events are more likely to appear. Such relationships are coherent with the social exchange theory and stress theory, according to which injustice creates anger, frustration and the feeling of being mistreated, which, in turn, increase deviant behaviors (Alias et al., 2013).

Organizational climate concerns the shared perceptions that employees have about organizational practices, procedures and behaviors that are expected and rewarded in the organization (Schneider et al., 1998). Narayanan and Murphy (2017) argue that employees will indulge in deviant behaviors when climate is strongly oriented to achieving organizational goals and ignoring employees’ wellbeing; however, a collectivistic organizational culture, that encourages belongingness, interdependence and attention to the needs of individuals, will moderate that relationship and will decrease the implementation of CWB. Manning (2020) distinguishes organizational climates in compliance-based and integrity-based. The first one describes an organization in which management systems and procedures encourage employees to comply with the practices just to avoid personal risks and regulatory sanctions, while in an integrity climate, management systems and procedures encourage behaviors based on those ethical values that are genuinely accepted by individuals and groups. In the first case, it is more probable to observe an ethical weakness that makes more probable the appearance of CWB and collective deviance because the organization requires or tolerates behaviors that reflect legislative compliance but, in essence, follows a least-cost logic. In integrity-based climates, the organization complies with moral and ethical standards and fights any cue of individual or collective CWB.

Work conditions, such as work stress and limited job autonomy, have also been proposed as possible causes of workplace deviant behaviors (Alias et al., 2013). A recent review on the effects of virtual offices underlines that interruptions, increased workload, and work-family conflict contribute to increased employee’s stress which, in turn, result in cyberloafing and cyberdeviance (Stich, 2020). The meta-analysis conducted by Sverke et al. (2019) on 106 studies concluded that job insecurity, conceptualized as the “perceived, unwelcome threat to the current job,” resulted slightly but positively associated with CWB (*r* = 0.14) (Sverke et al., 2019).

## Discussion

The present scoping review of reviews aimed to map the recent conceptual developments (1) about the deviant work behavior and counterproductive work behavior constructs, (2) about specific types of deviant work behavior that have been more consistently investigated in the last decade, and (3) the extent to which recent studies examine interpersonal and organizational types of deviant behavior. In addition, (4) we aimed to cover the available literature on the predictors of these types of behaviors. Considering the breadth of these research questions and the disparate literature about deviant behavior, a scoping review of reviews, summarizing research findings and establishing the value of undertaking a full systematic review, seemed the more suitable for this study.

Articles fitting our inclusion criteria were classified as “comprehensive” reviews when they examined the broad categories of DWB or CWB comprehending multiple types of deviant behaviors, and as “specific” reviews when examined only one specific type of deviant or counterproductive behavior, such as ostracism or incivility. Recent trends in this literature were observed and our results show that of the 54 retrieved reviews, published between 2010 and 2021, most fall under the “comprehensive” category (*n* = 36) and 18 under the “specific” one. This result seems to reflect a well alive effort to consider DWB and CWB as comprehensive constructs although with unclear and blurred borders. In fact, first of all, many authors, including us, continue to use the two terms interchangeably, which does not facilitate the delimitation of the boundaries of the field of study. Second, our study suggests that there is limited evidence of comprehensive models using new criteria to develop typologies to map the multiple manifestations of DWB or that extended previous typologies by incorporating new types of DWB or CWB. The classical models by Robinson and Bennett (1995), Gruys and Sackett (2003) or Spector et al. (2006) are still considered the starting point of much literature. The interpersonal vs. organizational distinction is mentioned in almost all papers that define and concern DWB and CWB, and thus can be considered as the basic characteristic, or *fundamentum divisionis*, to classify DWBs and CWBs. Some recent study described forms of deviance implemented by less examined actors, such as teams or organizations (Carpenter et al., 2020) or supplier and consumers (Lugosi, 2019); however, the main aim of these studies was not to arrange a classification of the forms of deviant work behaviors but to extend the DWB or CWB constructs. Finally, the hierarchical approach proposed by Sackett (2002) and further elaborated by Marcus et al. (2016) suggests a general latent factor subsuming middle-level constructs (such as theft or sabotage), which include, at the lower-level, the specific deviant behaviors. Unfortunately, this internal structure becomes psychometrically unstable when more CWB dimensions are included (Marcus et al., 2016). Thus, future studies on the hierarchical structure of the concept [that, according to Marradi (1990) corresponds to a taxonomy] are welcomed.

Third, some reviews examined CWB as a component of job performance (Koopmans et al., 2011; Ong, 2012; Campbell and Wiernik, 2015) while others examined CWB in relation to (un)ethical aspects. The first case, which extends the CWB construct toward the top of the hierarchical construct (CWB as one form of job performance) is more common in literature; the second case adds the criterion of un/ethical behavior to extend the types of behaviors subsumed by the CWB construct. Similarly, the meta-analysis by Carpenter and Berry (2017) provides evidence that withdrawal is a component, or a facet, of CWB, thus extending the reflection on behaviors included in the CWB construct. Fourth, the level of analysis is becoming a more consistent and rich issue, with some models examining multilevel antecedents of individual CWB (O’Boyle et al., 2011) and other models examining CWB performed by teams, departments or organizations and same-level antecedents (team antecedents of team CWBs, and so on). Fifth, a self-reinforcing mechanism should be better investigated because multiple reviews suggest that one type of DWB is the cause of other types of DWB. This was observed especially when considering specific types of DWB, e.g., abusive leadership, incivility or ostracism. The negative reciprocity theory (Faldetta, 2020) and especially the social exchange theory as proposed by Cropanzano et al. (2017) can provide useful suggestions to examine when the target reciprocates with the same type of deviant behavior performed by the actor (homeomorphic reciprocity) or when situational constraints suggest to reciprocate the offense with a different type of deviant behavior. Sixth, CWB measurement is another issue characterizing the field, with many scales measuring comprehensive CWB and specific CWB but with a shortage of objective measures. However, the evidence seems to suggest that self- vs. other-report measures of CWB provide comparable results (Berry et al., 2012). In sum, DWB and CWB constructs continue to attract attention and reflection by scholars, even if they remains blurred concepts whose internal structure, relations among types of negative work behaviors and relations with other external concepts remain not completely specified.

Our scoping review shows another interesting trend in CWB literature: only two reviews concerned an organizational type of CWB, namely cyberloafing, while the majority of the specific reviews examined interpersonal types of CWB. This seems an interesting result in itself because it is unclear if organizations developed effective measures (regulation, guidelines or policies) to better monitor and counteract organizational deviance, or if such type of deviance has taken (or is taking) other forms and other names. It is clear, anyway, that cyberloafing will become more relevant compared to other organizational CWB, such as theft or time banditry, considering that modern work will be more and more done remotely, especially after the experience of the COVID-19 pandemic. On the other hand, the abundant literature on the interpersonal CWBs reflects the increasing incidence of violence, aggression or basic uncivil conduct reported by employees in several organizations (Porath, 2016). Among these interpersonal CWBs, our review shows that ostracism stands out with the largest number of reviews. The growing interest in ostracism might be related to the fact that this behavior is highly ambiguous compared to other overt and more explicit interpersonal workplace abuse, such as bullying. Moreover, what distinguishes this interpersonal CWB is the lack of interaction between the perpetrator and the victim, in comparison to other forms of workplace mistreatment where both parties necessarily engage in a negative form of interaction (Ferris et al., 2017; Bedi, 2021).

Three main considerations can be advanced about antecedents of deviant behavior. First, the personological approach is very present, with many reviews focusing on socio-demographic and personality aspects of perpetrators and victims, such as dark triad, big five or Hexaco models (Wu and LeBreton, 2011; O’Boyle et al., 2012; Pletzer et al., 2020). Second, new approaches are emerging that take into account the influence that social and/or organizational contexts may have on individual deviant behavior. The role of the leader, not only as a perpetrator of destructive or abusive leadership but also as a key person that influences climate and establishes what is acceptable, rewarded or punished, is receiving more attention (O’Boyle et al., 2011; Tepper et al., 2017; Faldetta, 2020). The attention given to witnessed deviance goes in this direction: bystanders observe decisions taken by leaders and the organization, and are indirectly affected by the deviance experiencing a sense of injustice (Schilpzand et al., 2016; Dhanani and LaPalme, 2019). Multilevel impact on individual deviant behavior is just one aspect of this trend that is also going toward a closer examination of teams and organizations deviance (Carpenter et al., 2020). Third, it seems that despite the variety of deviant behaviors here considered, such behaviors share most of the antecedents. Studies on CWB in general, as well as ostracism, incivility, or abuse, share, at the various levels, most of the antecedents. Sharing antecedent across different types of deviant behaviors goes in the direction of a general latent factor, as suggested by Marcus et al. (2016); this is an issue that CWB scholars and future studies should examine more systematically.

### Limitations, Future Directions, and Recommendations for Practitioners

We believe that the scoping review approach used in this study was the most suitable approach to address such a broad domain and the broad research questions we posed. However, we are also aware that this kind of review method has several limitations. First, scoping reviews do not formally evaluate the quality of evidence and often gather information from a wide range of study designs and methods. Second, in scoping reviews the assessment of the risk of bias assessment is not mandatory, we are aware that we have not been very strict in assessing the methodological quality of the retrieved reviews. For instance, of the 54 included articles, the majority are non-systematic reviews (*n* = 33), while 14 studies were meta-analyses and seven were systematic reviews. Therefore, we reported some trends in the literature as well as solid evidence. Third, although we searched for “DWB or CWB” and “review or typology,” in the title, abstract and keywords, it is clear that the keyword typology was almost irrelevant. The keyword “meta-analysis” would have proved more effective. It is thus possible that some references of interest were not included in our list because they used a more figurative title, or never mentioned DWB, CWB or review, in the abstract or in keywords. We are at least aware of two meta-analyses, the one conducted by Carpenter and Berry (2017) and the one conducted by Greco et al. (2015) on the non-response bias in CWB. The former was anyway mentioned above, and the latter found that CWB studies report much lower response rates than that typically found in management research. We acknowledge here these studies, although we think their contributions do not compromise our conclusions. Fourth, because our interest was in the classification of negative deviant behaviors, we dropped records pertaining to positive deviant behaviors as well as five reviews proposing strategies to counteract CWB. Nonetheless, we believe that this scoping review provides a comprehensive and up to date overview of the different conceptualizations, approaches, typologies and predictors of DWB and CWB. We recommend practitioners to navigate the different sections of our review, especially the predictors section to get an idea of what variables they can control to limit the manifestations of such deviant behaviors. It is important to note, however, that practitioners should take in consideration their particular organizational context in order to better understand what may better work for them and what does not.

Concerning directions for future research, in addition to the various theoretical and methodological directions suggested above, some scholars have recently paved the way for some novel interesting perspectives on workplace incivility. As indicated by Schilpzand et al. (2016) and Irum et al. (2020), also Kabat-Farr et al. (2020) suggest that workplace incivility may be targeted toward employees who belong to devalued or stigmatized groups: ingroup and outgroup processes, gender composition, power and status, and cyber situations represent conditions in which specific individuals are more at risk of being mistreated. Therefore, future studies on CWB should take into account the aforementioned variables when investigating workplace incivility. In addition, considering that CWB studies have been traditionally focused on victims’ perspectives, future studies should investigate the impact and antecedents of CWB on witnesses (Schilpzand et al., 2016) or on the same actor of the CWB (Zhong and Robinson, 2021). Finally, as highlighted above, and in line with previous scholars (O’Boyle et al., 2011), a multilevel perspective should be taken into account when investigating CWB, be it ostracism, cyberloafing, or any other type through which CWB manifests itself.

## Author Contributions

SZ conducted the search queries and wrote and reviewed the manuscript. MYS contributed by integrating the search query, writing parts, and reviewing the final manuscript. All authors examined the retrieved reviews, reviewed, and helped shaping the manuscript.

## Conflict of Interest

The authors declare that the research was conducted in the absence of any commercial or financial relationships that could be construed as a potential conflict of interest.

## Publisher’s Note

All claims expressed in this article are solely those of the authors and do not necessarily represent those of their affiliated organizations, or those of the publisher, the editors and the reviewers. Any product that may be evaluated in this article, or claim that may be made by its manufacturer, is not guaranteed or endorsed by the publisher.
